# Sanbai Melon Seed Oil Exerts Its Protective Effects in a Diabetes Mellitus Model via the Akt/GSK-3*β*/Nrf2 Pathway

**DOI:** 10.1155/2019/5734723

**Published:** 2019-09-12

**Authors:** Fang Wang, Yuanhang Xi, Wenzhe Liu, Jie Li, Ya Zhang, Min Jia, Qiaoyan He, Hongsheng Zhao, Siwang Wang

**Affiliations:** ^1^Xi'an Siyuan University, 28 Shui An Road, Xi'an 710032, China; ^2^Department of Chinese Materia Medica and Natural Medicines, Air Force Medical University, Xi'an, 710032 Shaanxi, China; ^3^Yang Ling Fragrance Edible Oil Co. Ltd., 712100 Shaanxi Province, China; ^4^College of Life Science, Northwest University, 229 Taibai Beilu, Xi'an 710069, China; ^5^Shaanxi Key Laboratory of Ischemic Cardiovascular Disease, Institute of Basic and Translational Medical, Xi'an Medical College, 1 Xinwang road, Xi'an 710021, China

## Abstract

Traditional Chinese medicine (TCM) plays an important role in the treatment of type 2 diabetes mellitus (T2DM). However, the lack of adequate and scientifically rigorous evidence has limited its application in this disorder. Sanbai melon seed oil (SMSO) is used in folk medicine to treat DM; however, only few literature reports exist regarding its mechanism. Herein, we aimed to confirm the antidiabetic activity of SMSO in a T2DM model and further elucidate its possible mechanisms. The T2DM rat model was induced by high-fat and sugar diet and streptozocin (STZ, 40 mg/kg). SMSO was administered at doses of 0.7 g/kg, 1.4 g/kg, and 2.8 g/kg. Several biochemical parameters and antioxidant protein levels were measured to evaluate the hyperglycemic and antioxidant activities of SMSO. Western blotting was performed to determine its potential mechanism. Based on the results, SMSO treatment significantly reduced blood glucose levels, increased plasma insulin, and repaired islet tissue injury in diabetic rats (*P* < 0.05). To add, it markedly reduced MDA levels and increased that of catalase (CAT), superoxide dismutase (SOD), and glutathione peroxidase (GSH-Px). Western blot results showed that SMSO induced n-Nrf2 and HO-1 expression and Akt and GSK-3*β* phosphorylation in a dose-dependent manner. Further studies showed that LY294002, aPI3K inhibitor, abolished the effects of SMSO on GSK-3*β* phosphorylation and Nrf2 nuclear translocation as well as the protective effects on pancreatic *β* cells. Together, these results suggest that SMSO regulates the Akt/GSK-3*β*/Nrf2 pathway and induces the expression of antioxidant proteins to impede oxidative stress in rats with T2DM.

## 1. Introduction

Diabetes mellitus (DM) continues to function as a global and serious threat to the health of patients. Type 2 diabetes mellitus (T2DM) accounts for 90% of DM cases, and its prevalence is projected to rise to 366 million by 2030 [1]. Because of the increasing rates of obesity induced by unreasonable dietary patterns and poverty, the incidence of T2DM has remarkably increased in Southeast Asia and China [2]. T2DM and its associated complications have medical and socioeconomic burdens on health-care systems and impacts on economic wealth [3]. In clinics, several drugs, such as rosiglitazone and metformin, are used to treat T2DM. However, many reports have shown that synthetic drugs exhibit side effects in the treatment process [4]. Thus, the search for herbal agents to serve as better therapeutics is necessary for the treatment of T2DM.

The function islet *β* cells play an important role in the development of T2DM; its dysfunction leads to the onset of glucose intolerance, and its gradual worsening leads to unmet needs of long-term glycemic control [5, 6]. Some studies revealed that *β* cell dysfunction is closely linked to oxidative stress induced by hyperglycemia and hyperlipidemia [6]. In patients with T2DM, sustained hyperglycemia causes glucose autooxidation and protein glycosylation, which lead to excessive production of reactive oxygen species (ROS) [7]. The intrinsic antioxidant defense machinery is fragile in the *β* cell of these patients. Hence, pancreatic cells are susceptible to oxidative stress [8]. Antioxidants may therefore serve as a promising treatment strategy to avoid *β* cell dysfunction in T2DM.

In China, some fruits or vegetable oils are used as antioxidants to treat diseases [9]. *Citrullus lanatus*, also known as watermelon, is a type of dietetic fruit. The juice of *Citrullus lanatus* is delicious and is popular in many countries. Its seed contains many antioxidant compounds, such as tannins, flavonoids, terpenoids, and alkaloids, which have demonstrated antidiabetic effects in previous studies [10].

Citrullus lanatus cv. Sanbai (Citrullus lanatus (Thunb.) Matsum & Nakai cv. Sanbai) also known as Sanbai melon is a type of cultivated watermelon species in China. In folk medicine, Sanbai melon seed oil (SMSO) has displayed protective effects in DM. In our previous study, we found that the main constituents of SMSO were flavonoids and tocopherols, which displayed excellent antioxidant and antidiabetic activity in a T1DM model [11]. As there is no literature report on the effects and possible mechanisms of SMSO in a T2DM model, we aimed to determine the antidiabetic activity of SMSO in a T2DM model and elucidate its possible mechanisms.

## 2. Materials and Methods

### 2.1. Materials

Streptozotocin (STZ) and LY294002 were obtained from Sigma-Aldrich (St. Louis, MO, USA). Trisodium citrate and citric acid were obtained from Tianjin Hongyan Chemical Reagent Co. Ltd. (Tianjin, China). One-Touch Ultra Blood Glucose Meter and strips were purchased from Accu-Chek Performa, Roche, Germany. Malondialdehyde (MDA), glutathione peroxidase (GSH-Px), superoxide dismutase (SOD), and catalase (CAT) kits were purchased from Nanjing Jiancheng Science and Technology Co. Ltd. (Nanjing, China). Rat insulin ELISA kits were obtained from Sweden Mercodia Company (Sweden). Bax, Bcl-2, Nrf2, HO-1, AKT, GSK-3*β*, cleaved-caspase 3, caspase 3, Fyn, *β*-actin, and H3 primary antibodies were obtained from Cell Signaling Technology Inc. (Danvers, MA). Secondary antibodies were obtained from Cell Signaling Technology Inc. Other chemical reagents were of analytical grade.

### 2.2. Production of SMSO

Fresh Sanbai melons were obtained from Wanrong County, Yuncheng City, Shanxi Province, and botanically identified by Professor Wenzhe Liu, a botanist at the College of Life Sciences, Northwest University. The flesh and rinds were removed from the fruit, and its seeds were collected, washed, and sun-dried. Voucher specimens were deposited at the Department of Natural Medicine, School of Pharmacy, Air Force Medical University. Seed oil extractions were kindly performed by Yangling Piaoxiang Edible Oil Co. Ltd. Total flavonoid content was used to control oil quality.

### 2.3. Induction of T2DM

Male Sprague Dawley (SD) rats were obtained from the Experimental Animal Center of Air Force Medical University. All animal experimental protocols were performed according to the Guidelines for Animal Experimentation of Air Force Medical University and reviewed and approved by the Ethics Committee for Animal Experimentation (FMMU-201704067). Rats were housed in a controlled environment with a 12 h light-dark cycle at 25°C.

After adaptive feeding for 1 week, diabetic rats were fed a high-fat and sugar diet (lard oil 18%, sucrose 20%, yolk 3%, and basal feed 59%) [12], the rats in the control group (NC) were fed a basal diet, and rats in the SMSO control group (NC+SMSO) were fed a regular diet and 2.8 g/kg SMSO. After dietary manipulation for 4 weeks, diabetic rats were injected intraperitoneally with STZ (40 mg/kg BW, dissolved in sodium citrate buffer, pH 4.4), while rats in the control group were injected with vehicle citrate buffer with a volume of 1 mL/kg. After 3 days of administration, the level of fasting blood glucose (FBG) was measured via the tail vein in triplicate. Rats with an FBG ≥ 11.0 mmol/L were diagnosed with T2DM and were used for further studies.

Diabetes rats were divided into four groups: T2DM model group (Mod, *n* = 8), low-dose SMSO group (SMSO-L, 0.7 g/kg, *n* = 8), medium-dose SMSO group (SMSO-M, 1.4 g/kg, *n* = 8), and high-dose SMSO group (SMSO-H, 2.8 g/kg, *n* = 8). SMSO administration lasted 8 weeks. During the experimental period, body weight and FBG of rats were monitored weekly. At the end of 8 weeks, rats were sacrificed and blood samples were collected from the abdominal aorta. Whole blood was used to determine the level of glycosylated hemoglobin (HbA1c), and serum was used to determine the level of insulin and other biochemical parameters. Pancreatic tissues were also collected for further studies.

### 2.4. Histopathologic Examination

After blood collection, pancreatic tissues were excised carefully, washed with normal saline, fixed in 4% paraformaldehyde solution, and then embedded in paraffin. Each pancreas was cut into 5 *μ*m thick sections and then stained with hematoxylin-eosin staining (HE) for histopathological examination. Pathological changes were observed under an optical microscope.

### 2.5. Estimation of Antioxidant Levels

The levels of MDA, SOD, GSH-Px, and CAT were measured using commercial kits (Nanjing Jiancheng Science and Technology Co. Ltd.) with the provided instructions.

### 2.6. Immunohistochemistry

Tissue sections were prepared as per the method used above. After dewaxing, the sections were incubated in a water bath kettle oven at 98°C for 15 min with target retrieval solution for antigen retrieval, followed by treatment with 3% EDTA for 15 min and 5% BSA for 30 min. The sections were incubated with primary antibodies (Nrf2: 1 : 400, Cell Signaling Technology; HO-1: 1 : 400, Cell Signaling Technology) at 4°C overnight, washed with PBS, and incubated with horseradish peroxidase-conjugated secondary antibody (1 : 400, Cell Signaling Technology) for 1.5 h at room temperature. For color development, sections were treated with the peroxidase substrate, DAB, and counterstained with hematoxylin (Boster Biological Technology Co. Ltd.).

### 2.7. Immunoblot Analysis

Pancreatic tissues were homogenized with pancreas lysis buffer (7 mol/L carbamide, 2 mol/L thiocarbamide, 5 mL/L IPG buffer (pH 3-10), 65 mmol/L DTT, 20 g/L octanoyl thioglycine trimethyl salt (SB 3-10), 20 g/L CHAPS, 5 mg/L proteinase inhibitor, and 10 mL/L trypsin inhibitor) [13]. Nuclear proteins and cytoplasmic proteins were isolated using a commercial kit from Baiaolaibo Co. Ltd. (Beijing, China). Protein concentration was determined with a BCA protein assay kit (Roche). For western blot analysis, an equal amount of protein (30 *μ*g) from each sample was separated by 10% SDS-PAGE followed by transferal to a polyvinylidene fluoride membrane (Millipore). Membranes were then blocked with 5% nonfat dried milk at room temperature for 1 h and then incubated with primary antibodies at 4°C overnight. The following primary antibodies: anti-P-GSK-3*β* (1 : 1000), anti-GSK-3*β* (1 : 1000), anti-Nrf2 (1 : 500), anti-HO-1 (1 : 1000), anti-caspase 3 (1 : 1000), anti-Bcl2 (1 : 1000), anti-Bax (1 : 1000), anti-P-Akt (1 : 500), anti-Akt (1 : 500), anti-Fyn (1 : 500), anti-*β*-actin (1 : 1000), and anti-H3 (1 : 1000), were obtained from Cell Signaling Technology. After washing with Tris-buffered saline; membranes were incubated with horseradish peroxide-conjugated secondary antibodies at room temperature for 1 h. Membranes were visualized using an enhanced chemiluminescence (ECL) detection kit (Millipore). *β*-Actin was used as protein loading control for the cytoplasm while H3 was used as protein loading control for the nucleus.

### 2.8. Statistical Analysis

Statistical analysis was performed with GraphPad Prime 5.0 software. All data are expressed as the mean ± SD. Comparisons between groups were analyzed by the one-way analysis of variance (ANOVA) and Student-Newman-Keuls test. A *P* value< 0.05 was considered statistically significant.

## 3. Results

### 3.1. General Characteristics of Diabetic Rats after SMSO Treatment

**Figure 1:**
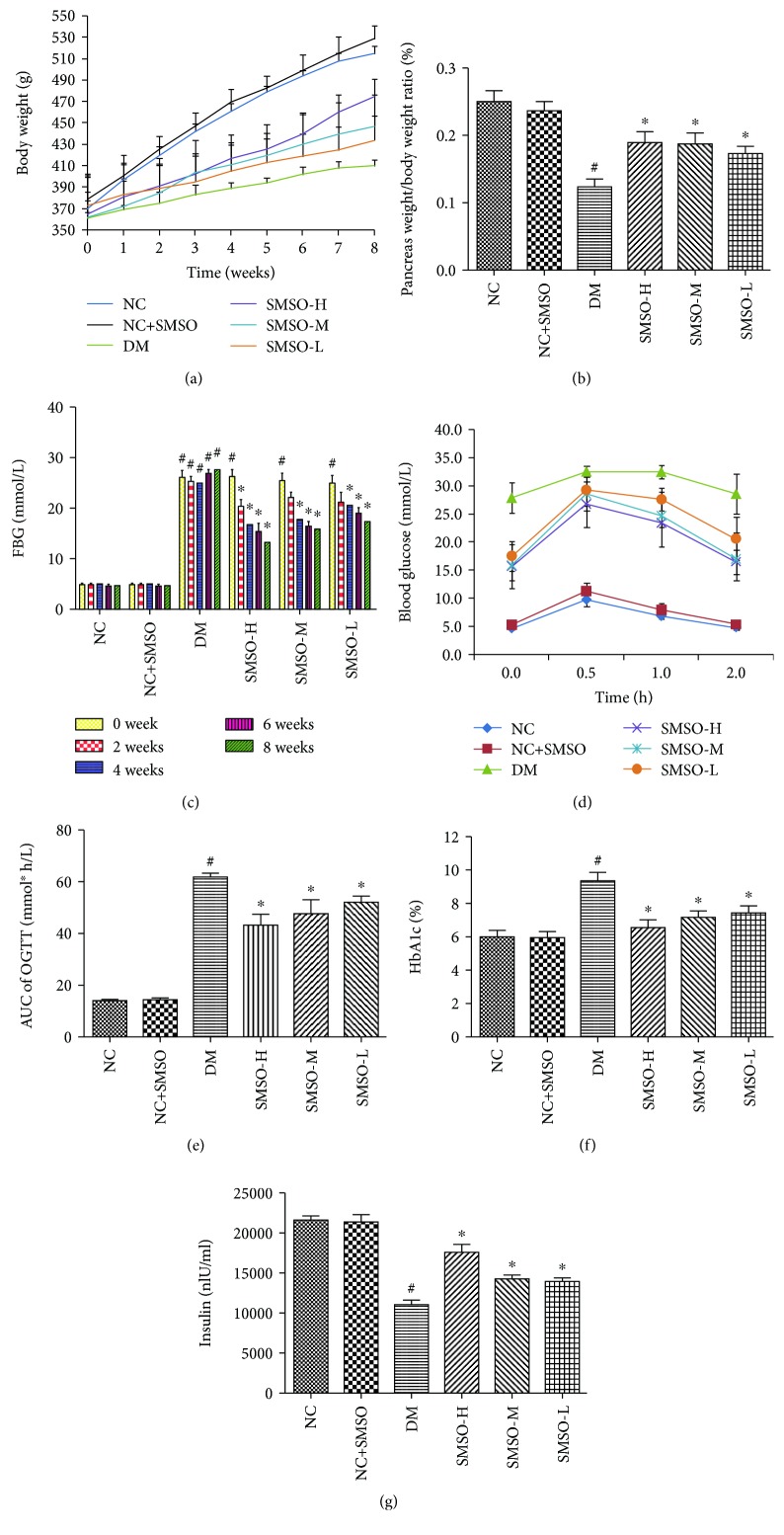
Effects of SMSO on the general characteristics of diabetic rats. (a) Effects of SMSO on body weight gain in diabetic rats every week after SMSO treatment. (b) The pancreas weight/body weight ratio was measured at the end of the experiment. (c) Effects of SMSO on FBG levels in diabetic rats at 0, 2, 4, 6, and 8 weeks after SMSO treatment. (d) Effects of SMSO on oral glucose tolerance at the end of the experiment. (e) AUC of oral glucose tolerance results. (f) Effects of SMSO on HbA1c levels in diabetic rats. (g) Effects of SMSO on plasma insulin levels in diabetic rats. ^#^*P* < 0.05 vs. the NC group. ^∗^*P* < 0.05 vs. the DM group.

Body weight was recorded every week while FBG was recorded every two weeks. As shown in Figure 1(a), body weights in all groups were gradually increased. At the end of the experiment, body weight in the DM group was lower than that in the NC group; however, treatment with SMSO restored the loss in body weight. The pancreas weight/body weight ratio in the DM group was significantly lower than that in the NC group (*P* < 0.05); however, SMSO treatment could significantly increase the ratio in a dose-dependent manner (Figure 1(b)).

As shown in Figure 1(c), from treatment initiation to the 8^th^ week, a higher level of FBG was found in the DM group than in the NC group. However, SMSO treatment could significantly lower this level in a dose-dependent manner (*P* < 0.05). The results of the oral glucose tolerance test showed that blood glucose levels in each group were increased at 0.5 h after the administration of 50% glucose solution [14] and were restored to normal levels at 1 h (Figure 1(d)). The blood glucose level in the DM group was higher than that in the NC group, suggesting that pancreatic islet function was destroyed. The AUC results showed that SMSO treatment significantly restored the function of the pancreatic islet (Figure 1(e)). In the DM group, the HbA1c level was significantly higher than that found in the NC group; however, the insulin level was significantly lower than that found in the NC group (*P* < 0.05). SMSO treatment significantly decreased the HbA1c level and increased the insulin level in a dose-dependent manner (Figures 1(f) and 1(g)).

### 3.2. Effects of SMSO on Histological Changes

**Figure 2:**
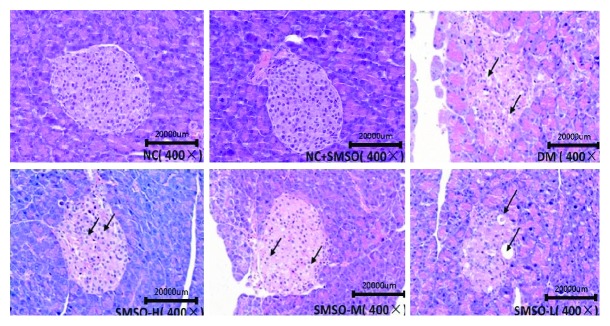
Effects of SMSO on pancreatic histological changes. Histological changes were evaluated by HE staining. Original magnification: 400x.

Figure 2 shows the histopathological results of the pancreas of rats. The islet cells had a normal appearance in the NC group, but in the DM group, a decrease in the size of pancreatic islets was found. Verges were ambiguous, cells were undergoing karyopyknosis and degranulation, and vacuolation and invasion of connective tissues were detected. In the SMSO group, the islet cells were saved and restored in a dose-dependent manner while the Langerhans cells and size of the islet in the high-dose group were restored to levels near those found in the NC group. No such changes were found in normal rats treated with SMSO.

### 3.3. Effects of SMSO on Apoptosis

**Figure 3:**
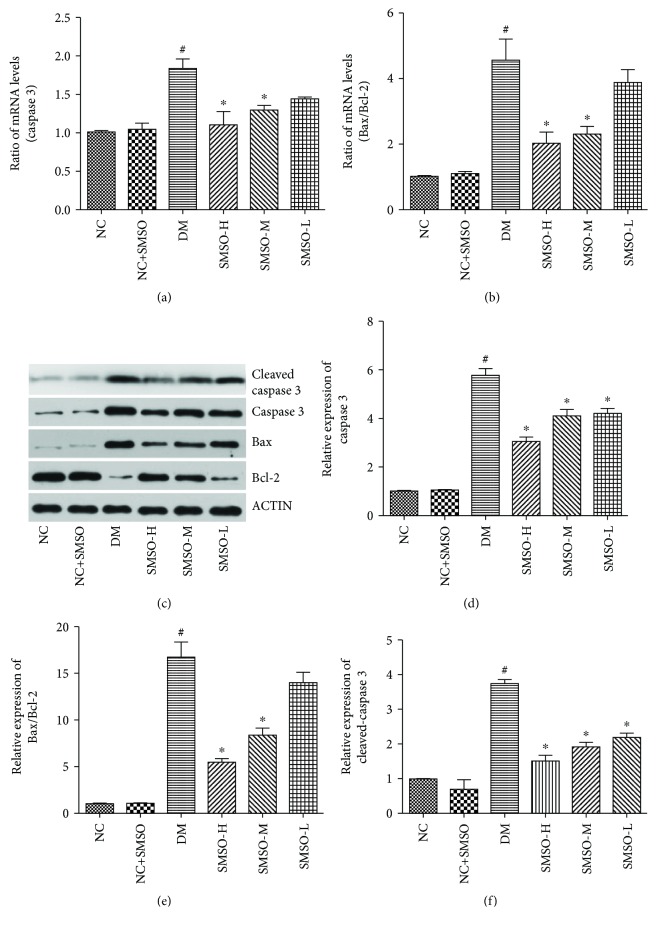
Effects of SMSO on pancreatic apoptosis. Apoptosis-related protein levels (caspase 3, Bax, and Bcl-2) in pancreatic tissues after different treatments were measured by RT-PCR and western blot analysis. ^#^*P* < 0.05 vs. the NC group. ^∗^*P* < 0.05 vs. the DM group.

To determine whether SMSO had antiapoptotic effects during the treatment process, caspase 3, Bax, and Bcl-2 were measured by RT-PCR and western blot analysis. mRNA levels of caspase 3 and the ratio of Bax/Bcl-2 were significantly increased in the DM group compared to the NC group (Figures 3(a) and 3(b)). The protein expressions of cleaved-caspase 3, caspase 3, and the ratio of Bax/Bcl-2 were the same as the levels of mRNA (Figures 3(c)–3(e)). SMSO treatment was found to inhibit the mRNA and protein expression of caspase 3 and Bax and significantly increased the expression of Bcl-2, ultimately demonstrating its antiapoptotic property.

### 3.4. Effects of SMSO on Antioxidant Status

**Figure 4:**
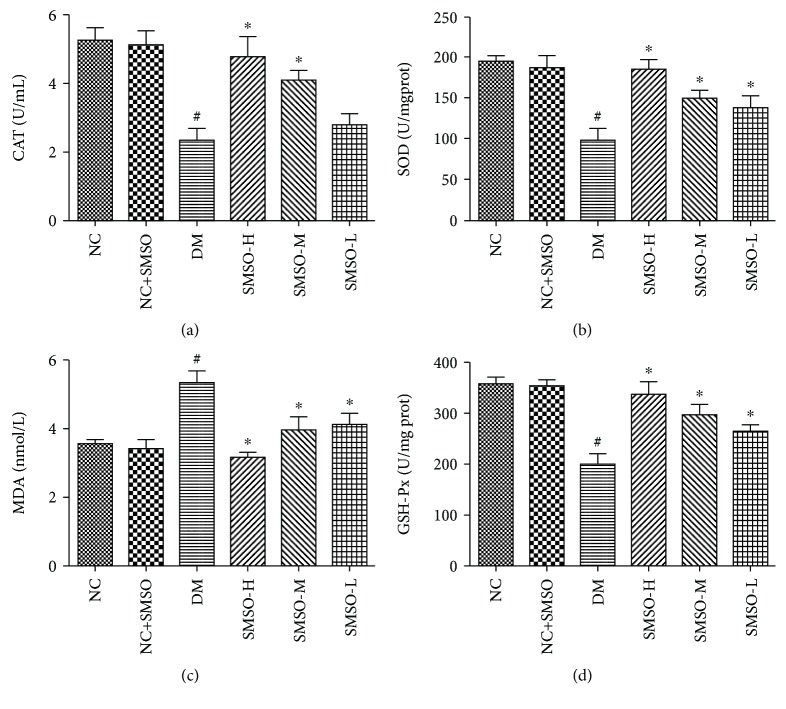
Effects of SMSO on the level of oxidative stress. (a) CAT levels in diabetic rats after SMSO treatment. (b) SOD levels in diabetic rats after SMSO treatment. (c) MDA levels in diabetic rats after SMSO treatment. (d) GSH-Px levels in diabetic rats after SMSO treatment. ^#^*P* < 0.05 vs. the NC group. ^∗^*P* < 0.05 vs. the DM group.

Long-term hyperglycemia induces oxidative stress in the islet cells. To determine whether SMSO had antioxidative effects, we measured the levels of MDA, SOD, GSH-Px, and CAT. As shown in Figure 4, MDA levels were significantly increased while those of SOD, GSH-Px, and CAT were significantly decreased in the DM group (*P* < 0.05). By administering SMSO to animals, these changes were altered as values were restored to levels near normal. Such findings indicate that SMSO provides protection against hyperglycemia-induced oxidative stress.

### 3.5. Effects of SMSO on the Expression of Nrf2 in Diabetic Rats

**Figure 5:**
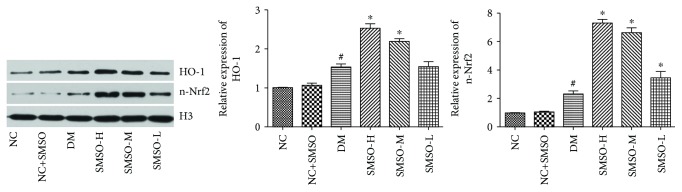
Effects of SMSO on the Nrf2 pathway. Immunoblotting of nuclear protein extracts from pancreatic tissues of diabetic rats. Representative bands of western blotting. Statistical data were derived from gray analysis. ^#^*P* < 0.05 vs. the NC group. ^∗^*P* < 0.05 vs. the DM group.

**Figure 6:**
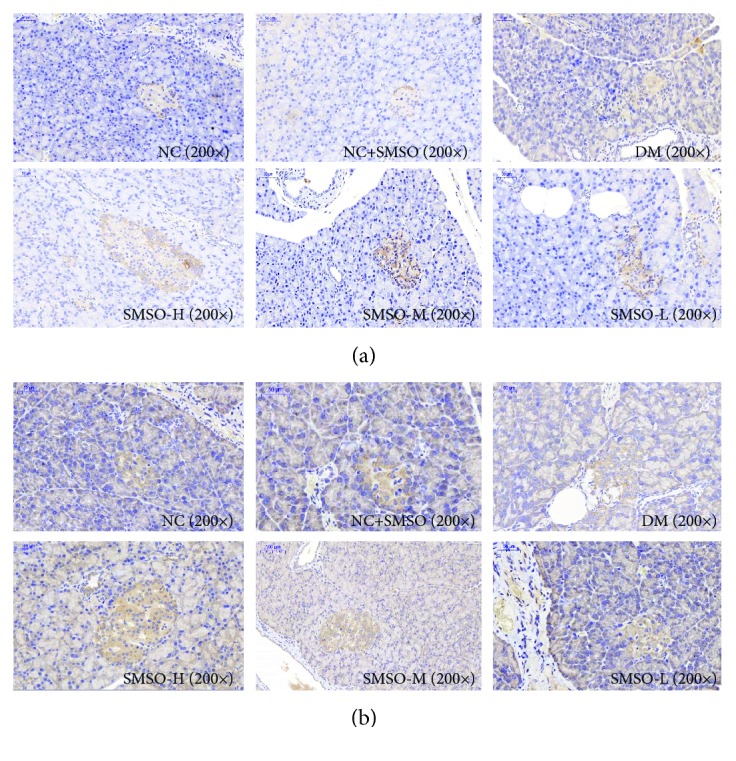
Effects of SMSO on the expression of Nrf2 and HO-1. (a) Immunohistochemical photograph of Nrf2 in the pancreatic tissues of different groups as indicated horizontally. (b) Immunohistochemical photograph of HO-1 in the pancreatic tissues. Original magnification: 200x.

Protein expressions of antioxidants are regulated via the Nrf2 pathway. Hence, to investigate whether the Nrf2 pathway might be responsible for the protective effect of SMSO, the expression levels of n-Nrf2 and HO-1 were measured by western blot analysis. As shown in Figure 5, DM induced the protein expression of n-Nrf2 and HO-1 compared to that observed in the NC group. Treatment with SMSO could significantly increase n-Nrf2 and HO-1 expression levels relative to that found in the DM group. Immunohistochemical results presented in Figures 6(a) and 6(b) reveal that SMSO induced the expression of Nrf2 and HO-1 in pancreatic tissues. The results were similar to those found by western blot analysis and indicate that SMSO could regulate the Nrf2 pathway, which induces antioxidant protein expression.

### 3.6. Effects of SMSO on the Activities of Akt and GSK-3*β*

**Figure 7:**
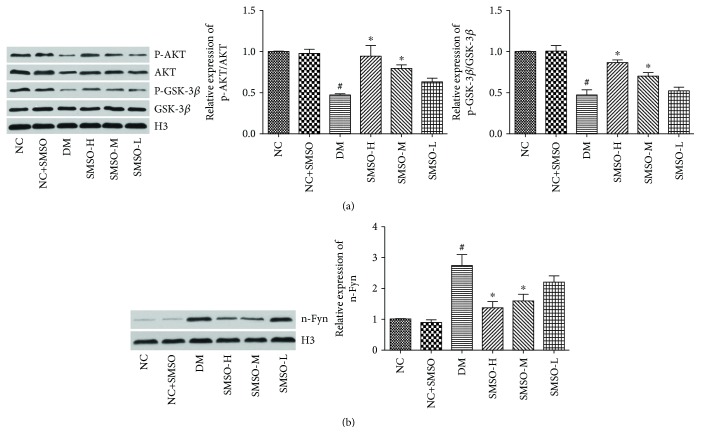
Effects of SMSO on the phosphorylation levels of Akt and GSK-3*β* in diabetic rats. (a) The phosphorylation levels of Akt and GSK-3*β* were determined by western blotting. (b) Expression levels of n-Fyn. ^#^*P* < 0.05 vs. the NC group. ^∗^*P* < 0.05 vs. the DM group.

To determine whether Akt and GSK-3*β* are involved in the protective effects of SMSO, the phosphorylated forms of Akt and GSK-3*β* were assessed by western blotting. As shown in Figure 7(a), the phosphorylation levels of Akt and GSK-3*β* were significantly decreased in the DM group. However, after treatment with SMSO, these levels were significantly increased in a dose-dependent manner (Figure 7(a)). The expression level of Fyn in the nucleus was also assessed. As shown in Figure 7(b), this level was significantly induced in the DM group; however, when treated with SMSO, the expression decreased in a dose-dependent manner.

### 3.7. Akt Pathway Is Necessary for SMSO-Induced GSK-3*β* Phosphorylation and Nrf2 Nuclear Translocation

**Figure 8:**
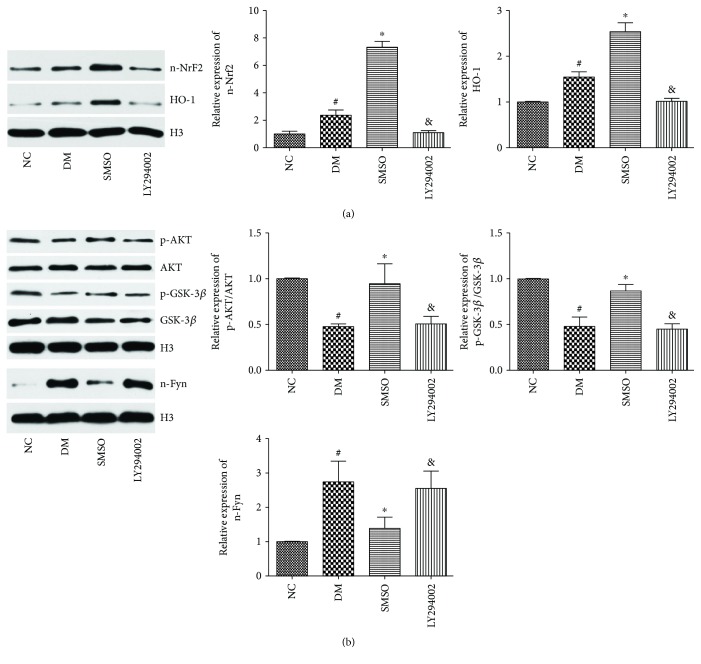
The Akt pathway is necessary for SMSO-induced GSK-3*β* phosphorylation and Nrf2 nuclear translocation. LY294002 and SMSO were administered to rats via tail vein intravenous injection (0.5 mg/kg, weekly). Islet tissues were then collected for western blot analysis. (a) Protein expression level of n-Nrf2 and HO-1 in cells. (b) Phosphorylation of Akt and GSK-3*β* and the expression level of n-Fyn were determined by western blotting. ^#^*P* < 0.05 vs. the NC group. ^∗^*P* < 0.05 vs. the DM group. ^&^*P* < 0.05 vs. the SMSO treatment group.

To determine whether Akt activation is necessary for SMSO-mediated GSK-3*β* phosphorylation and Nrf2 nuclear translocation, diabetic rats were treated with SMSO with or without pretreatment with LY294002. As shown in Figure 8, Akt and GSK-3*β* phosphorylation induced by SMSO and the expression levels of n-Nrf2 and HO-1 were inhibited by LY294002. The inhibitory effect of SMSO on the nuclear expression levels of n-Fyn was abolished by LY294002 treatment. These results suggest that the Akt pathway is necessary for SMSO-induced GSK-3*β* phosphorylation and Nrf2 nuclear translocation.

## 4. Discussion

DM is induced by the destruction or dysfunction of the pancreatic *β* cells and is considered to be one of the most influential metabolic diseases on human health [15]. Although dysfunction of pancreatic *β* cells plays important roles in the onset and development of DM, only few of the antidiabetics used in clinics improve *β* cell function [5]. Several reports revealed that pancreatic *β* cell dysfunction leads to an incremental increase in blood glucose, sustained hyperglycemia-induced oxidative stress, and other cellular stresses that further cause pancreatic *β* cell dysfunction [16]. Hence, a drug possessing hypoglycemic and antioxidant properties would serve as a useful treatment for DM. However, satisfactory outcomes have not been achieved with currently used approaches, prompting the development of new alternative approaches. Traditional Chinese medicine (TCM) has been used for thousands of years due to its accessibility, low cost, efficacy, and simplicity [17–19]. Under most conditions, TCMs have been used as empirical medicines, but adequate scientific evidence regarding their mechanism has not been gathered [19–21]. SMSO has been used in folk medicine to treat DM; however, limited research has been performed to verify its efficacy as an antidiabetic agent and its possible beneficial role in protecting pancreatic *β* cells. The present study was performed to investigate the effect of SMSO and further clarify its possible mechanism.

A high-fat and sugar diet combined with STZ is regularly used to induce the type 2 diabetic rat model. This is because it can mimic the metabolic features of T2DM in humans [22]. Chronic consumption of a high-fat and sugar diet can induce insulin resistance in rats, and STZ injection can destroy the pancreatic *β* cells, thereby mimicking the natural disease process from insulin resistance to *β* cell dysfunction [23]. In the present study, levels of glucose and HbA1c were significantly increased, and these were accompanied by a decrease in insulin levels in the DM group compared to normal rats. Administering SMSO induced dose-dependent changes in these parameters, and treatment with a high dose of SMSO exerted the best effects. Subsequent histological results showed that islet shrinkage, cellular swelling, and islet tissue vacuolation were observed in the pancreatic sections of DM rats. SMSO treatment alleviated the destruction in the islet caused by STZ. These findings indicate that SMSO ameliorated injury in the pancreatic islet and decreased blood glucose levels in a T2DM rat model, suggesting its antidiabetic activity.

During T2DM, persistent hyperglycemia induces the overproduction of ROS in cells. ROS could directly or indirectly destroy the normal physiological functions of cells by impairing DNA, protein, and other signaling pathways [24]. When cells are exposed to ROS, they develop an unexpected adaptive protection to counteract excess ROS through the increased production of antioxidant proteins [25]. However, persistent elevation or prolonged exposure reduced antioxidant levels and impaired the prooxidant/antioxidant balance [26]. Thus, antioxidants might be useful in the treatment of T2DM. Flavonoids and tocopherols have been identified as the main components of SMSO, which exerted an excellent antioxidant activity and have been used as a treatment for DM [11]. SMSO may also have some effects on ROS accumulation or antioxidant production. Interestingly, we observed that SMSO decreased MDA levels and increased SOD, GSH-Px, and CAT levels in T2DM rats, suggesting that SMSO could induce the production of antioxidant proteins to clear excess ROS. ROS induces apoptosis in cells which is the main reason for the loss of *β* cells in the pancreatic islet [27]. In this study, cleaved-caspase 3, caspase 3, and Bax were found to significantly increase in the DM group. However, by treatment with SMSO, their levels could be significantly decreased while the expression of Bcl-2 increased. These findings suggest that SMSO could inhibit cell apoptosis caused by oxidative stress.

To identify the potential molecular mechanism revealing SMSO as an antioxidant agent, we examined the Nrf2 pathway. Nrf2 induces antioxidant protein expression and plays an important role in inhibiting oxidative stress [28]. In normal conditions, Nrf2 resides in the cytosol with keap1. Once cells are subjected to stress, such as ROS, hyperglycemia, or ER stress, Nrf2 dissociates from keap1 and is translocated to the nucleus, thereby inducing various antioxidant gene expressions [29]. Nrf2 protects *β* cells from cell injuries and apoptosis induced by oxidative stress, ultimately minimizing the impairment in insulin secretion [30]. In this study, we found that STZ treatment induced a slight elevation in n-Nrf2 and HO-1 expressions; however, by administering SMSO, the levels of n-Nrf2 and HO-1 were significantly increased. Akt and GSK-3*β* were found to be the upstream molecular markers of Nrf2 in various cells [31]. In addition, some researchers found that GSK-3*β* could inhibit nuclear translocation of Nrf2 [32]. To determine the effects of SMSO on Nrf2 whether through Akt and GSK-3*β*, the levels of Akt and GSK-3*β* phosphorylation were measured. This revealed that SMSO could induce the phosphorylation of Akt and GSK-3*β* in a dose-dependent manner. To further verify whether the protective effects of SMSO occurred via Akt, we employed the PI3K/Akt inhibitor, LY294002. The results showed that LY294002 abolished the effects of SMSO on GSK-3*β* phosphorylation and Nrf2 nuclear translocation, as well as its protective effects on pancreatic tissues. These results suggest that SMSO regulated the Akt/GSK-3*β*/Nrf2 pathway and then induced the expression of antioxidant proteins to impede high glucose-induced oxidative stress.

## 5. Conclusion

To summarize, the results presented herein demonstrate that SMSO significantly reduced blood glucose levels, increased plasma insulin, repaired islet tissue injury, and increased the antioxidant activities in diabetic rats, ultimately verifying its excellent antidiabetic effect. The mechanism of SMSO may occur through the induction of Nrf2 via the Akt/GSK-3*β*-mediated pathway, which would induce the expression of antioxidant proteins to impede the oxidative stress induced by high glucose in rats with T2DM.

## Data Availability

The all data used to support the findings of this study are available from the corresponding author upon request.

## References

[1] Wild S., Roglic G., Green A., Sicree R., King H. (2004). Global prevalence of diabetes: estimates for the year 2000 and projections for 2030. *Diabetes Care*.

[2] Gu D., Reynolds K., Wu X. (2005). Prevalence of the metabolic syndrome and overweight among adults in China. *Lancet*.

[3] Stumvoll M., Goldstein B. J., van Haeften T. W. (2008). Type 2 diabetes: pathogenesis and treatment. *Lancet*.

[4] Chiang C.-K., Ho T. I., Peng Y. S. (2007). Rosiglitazone in diabetes control in hemodialysis patients with and without viral hepatitis infection effectiveness and side effects. *Diabetes Care*.

[5] Sjöholm Å. (2010). Liraglutide therapy for type 2 diabetes: overcoming unmet needs. *Pharmaceuticals*.

[6] Drews G., Krippeit-Drews P., Düfer M. (2010). Oxidative stress and beta-cell dysfunction. *Pflügers Archiv - European Journal of Physiology*.

[7] Koeck T., Corbett J. A., Crabb J. W., Stuehr D. J., Aulak K. S. (2009). Glucose-modulated tyrosine nitration in beta cells: targets and consequences. *Archives of Biochemistry and Biophysics*.

[8] Lenzen S., Drinkgern J., Tiedge M. (1996). Low antioxidant enzyme gene expression in pancreatic islets compared with various other mouse tissues. *Free Radical Biology & Medicine*.

[9] Liu C., Zhao Y., Li X., Jia J., Chen Y., Hua Z. (2014). Antioxidant capacities and main reducing substance contents in 110 fruits and vegetables eaten in China. *Food and Nutrition Sciences*.

[10] Fatema K., Habib B., Afza N., Ali L. (2003). Glycemic, non-esterified fatty acid (NEFA) and insulinemic responses to watermelon and apple in type 2 diabetic subjects. *Asia Pacific Journal of Clinical Nutrition*.

[11] Wang F., Li H., Zhao H. (2018). Antidiabetic activity and chemical composition of *Sanbai* melon seed oil. *Evidence-Based Complementary and Alternative Medicine*.

[12] Islam M. S., Choi H. (2009). Antidiabetic effect of Korean traditional *Baechu* (Chinese cabbage) kimchi in a type 2 diabetes model of rats. *Journal of Medicinal Food*.

[13] Dang J., Cai W., Li B. (2008). Mass chromatographic analysis of two methods of total protein extraction from pancreatic tissue. *Chinese Journal of General Surgery*.

[14] Kochikuzhyil B. M., Devi K., Fattepur S. R. (2010). Effect of saturated fatty acid-rich dietary vegetable oils on lipid profile, antioxidant enzymes and glucose tolerance in diabetic rats. *Indian Journal of Pharmacology*.

[15] Liu Y., Liu S. X., Cai Y., Xie K. L., Zhang W. L., Zheng F. (2015). Effects of combined aerobic and resistance training on the glycolipid metabolism and inflammation levels in type 2 diabetes mellitus. *Journal of Physical Therapy Science*.

[16] Bhattacharya S., Manna P., Gachhui R., Sil P. C. (2011). D-Saccharic acid-1,4-lactone ameliorates alloxan-induced diabetes mellitus and oxidative stress in rats through inhibiting pancreatic beta-cells from apoptosis via mitochondrial dependent pathway. *Toxicology and Applied Pharmacology*.

[17] Jia W., Gao W., Tang L. (2003). Antidiabetic herbal drugs officially approved in China. *Phytotherapy Research*.

[18] Ning G., Hong J., Bi Y. (2009). Progress in diabetes research in China. *Journal of Diabetes*.

[19] Tyler V. E. (2000). Herbal medicine: from the past to the future. *Public Health Nutrition*.

[20] Larussa T., Oliverio M., Suraci E. (2017). Oleuropein decreases cyclooxygenase-2 and interleukin-17 expression and attenuates inflammatory damage in colonic samples from ulcerative colitis patients. *Nutrients*.

[21] Ogunwande I. A., Avoseh O. N., Olasunkanmi K. N., Lawal O. A., Ascrizzi R., Flamini G. (2019). Chemical composition, anti-nociceptive and anti-inflammatory activities of essential oil of *Bougainvillea glabra*. *Journal of Ethnopharmacology*.

[22] Srinivasan K., Viswanad B., Asrat L., Kaul C. L., Ramarao P. (2005). Combination of high-fat diet-fed and low-dose streptozotocin-treated rat: a model for type 2 diabetes and pharmacological screening. *Pharmacological Research*.

[23] Zhang F., Ye C., Li G. (2003). The rat model of type 2 diabetic mellitus and its glycometabolism characters. *Experimental Animals*.

[24] Evans J. L., Goldfine I. D., Maddux B. A., Grodsky G. M. (2003). Are oxidative stress-activated signaling pathways mediators of insulin resistance and *β*-cell dysfunction?. *Diabetes*.

[25] Nguyen T., Sherratt P. J., Pickett C. B. (2003). Regulatory mechanisms controlling gene expression mediated by the antioxidant response element. *Annals of the New York Academy of Sciences*.

[26] Dröge W. (2002). Free radicals in the physiological control of cell function. *Physiological Reviews*.

[27] Kikuta K., Masamune A., Hamada S., Takikawa T., Nakano E., Shimosegawa T. (2013). Pancreatic stellate cells reduce insulin expression and induce apoptosis in pancreatic *β*-cells. *Biochemical and Biophysical Research Communications*.

[28] Li H., Wang F., Zhang L. (2011). Modulation of Nrf2 expression alters high glucose-induced oxidative stress and antioxidant gene expression in mouse mesangial cells. *Cellular Signalling*.

[29] Cullinan S. B., Diehl J. A. (2004). PERK-dependent activation of Nrf2 contributes to redox homeostasis and cell survival following endoplasmic reticulum stress. *Journal of Biological Chemistry*.

[30] Pi J., Zhang Q., Fu J. (2010). ROS signaling, oxidative stress and Nrf2 in pancreatic beta-cell function. *Toxicology and Applied Pharmacology*.

[31] Li C., Pan Z., Xu T., Zhang C., Wu Q., Niu Y. (2014). Puerarin induces the upregulation of glutathione levels and nuclear translocation of Nrf2 through PI3K/Akt/GSK-3*β* signaling events in PC12 cells exposed to lead. *Neurotoxicology and Teratology*.

[32] Tomobe K., Shinozuka T., Kuroiwa M., Nomura Y. (2012). Age-related changes of Nrf2 and phosphorylated GSK-3*β* in a mouse model of accelerated aging (SAMP8). *Archives of Gerontology and Geriatrics*.

